# Sebelipase alfa enzyme replacement therapy in Wolman disease: a nationwide cohort with up to ten years of follow-up

**DOI:** 10.1186/s13023-021-02134-3

**Published:** 2021-12-14

**Authors:** Tanguy Demaret, Florence Lacaille, Camille Wicker, Jean-Baptiste Arnoux, Juliette Bouchereau, Claire Belloche, Cyril Gitiaux, David Grevent, Christine Broissand, Dalila Adjaoud, Marie-Thérèse Abi Warde, Dominique Plantaz, Soumeya Bekri, Pascale de Lonlay, Anaïs Brassier

**Affiliations:** 1 Pediatric Department, Cliniques universitaires Saint-Luc, Université Catholique de Louvain (UCLouvain), Brussels, Belgium; 2 Gastroenterology-Hepatology-Nutrition Unit, Hôpital Necker-Enfants Malades, Assistance Publique Hôpitaux de Paris (APHP), Université de Paris, Paris, France; 3 Reference Center for Inherited Metabolic Diseases, Hôpital Necker-Enfants Malades, Assistance Publique Hôpitaux de Paris (APHP), Institut Imagine, Université de Paris, 149 Rue de Sèvres, 75015 Paris, France; 4 Paediatric Neurophysiology Department and Reference Center for Neuromuscular Diseases, Hôpital Necker-Enfants Malades, Assistance Publique Hôpitaux de Paris (APHP), Université de Paris, Paris, France; 5 Paediatric Radiology Department, Hôpital Necker Enfants Malades, Assistance Publique Hôpitaux de Paris (APHP), Université de Paris, Paris, France; 6 Pharmacy Department, Hôpital Necker Enfants Malades, Assistance Publique Hôpitaux de Paris (APHP), Université de Paris, Paris, France; 7 Pediatric Oncology and Hematology Department, CHU Grenoble Alpes, Grenoble, France; 8 Pediatric Neurology Department, CHU de Strasbourg, Strabourg, France; 9 Metabolic Biochemistry Department, CHU de Rouen, INSERM U1245, Université de Rouen Normandie, Rouen, France; 10 Centre for Human Genetics, Institut de Pathologie et de Génétique, Gosselies, Belgium

**Keywords:** Fatty liver disease, Lysosomal storage disease, Myopathic phenotype, Health-related quality of life, Rapidly progressive lysosomal acid lipase deficiency

## Abstract

**Background:**

Wolman disease (WD), the rapidly progressive phenotype of lysosomal acid lipase (LAL) deficiency, presents in neonates with failure to thrive and hepatosplenomegaly, and leads to multi-organ failure and death before 12 months of age. In clinical trials, enzyme replacement therapy (ERT) with sebelipase alfa led to improved survival, growth and biological parameters in WD patients followed up to 5 years. Long-term follow-up and health-related quality of life (HRQoL) evaluation are lacking.

**Results:**

We performed a nationwide, retrospective study of sebelipase alfa in WD patients. Five patients with abolished LAL activity and bi-allelic *LIPA* mutations were included with a median follow-up of 7 years (1–10). ERT was initiated at a median age of 1 month (0–4). Infusion tolerance was excellent on the long-term with only one patient requiring systematic pre-medication. Cholestyramine, fat-soluble vitamin supplements and a specific diet (high in medium-chain triglycerides and low in long-chain fatty acids) were prescribed. Liver function tests, plasma lipid profiles, fat-soluble vitamin levels and growth parameters improved. Three patients transiently exhibited a neuromyopathic phenotype (footdrop gait, waddling walk or muscle fatigue) but electromyography and muscle strength testing were normal. At last follow-up, all patients were alive with normal growth parameters and a satisfactory HRQoL, no patient had special education needs, and one patient required parenteral nutrition since an acute gastroenteritis.

**Conclusions:**

Early ERT initiation allowed 100% survival with positive outcomes. Very long-term follow-up and hematopoietic stem cell transplantation while on ERT should be evaluated to strengthen the benefits of sebelipase alfa.

## Background

Lysosomal acid lipase (LAL, EC 3.1.1.13) deficiency (LALD, MIM 278000) is an ultrarare lysosomal storage disease (LSD) associated with bi-allelic *LIPA* pathogenic variants causing strongly decreased LAL activity [1]. LAL hydrolyzes cholesteryl esters and triglycerides in lysosomes. Its deficiency leads to intra-cellular lipids accumulation especially in the liver, spleen, lymph nodes, intestine and bone marrow [2, 3].

LALD phenotypes form a continuous spectrum between Wolman disease (WD) [4, 5] and cholesterol ester storage disease (CESD). WD, also known as rapidly progressive LALD, has a low birth prevalence (1 per 1,000,000) [6]. WD patients present in the first months of life with failure to thrive, malabsorption, hepatosplenomegaly, liver failure and bilateral adrenal calcifications [7]. Without treatment, the children die from multi-organ failure within the first year of life (median: 3.7 months [8]). CESD has a higher prevalence of 1 per 160,000 [9], and presents during childhood or adulthood with hepatomegaly, elevated liver enzymes and dyslipidemia [10].

Liver transplantation (LT) or hematopoietic stem cell transplantation (HSCT) were the first treatments evaluated for WD, but the results were disappointing [11–15]. In 2015, sebelipase alfa, a recombinant human LAL enzyme replacement therapy (ERT), was granted a marketing authorization for the treatment of LALD. A phase 2/3 open-label clinical trial (LAL-CL03) evaluated sebelipase alfa efficacy on the survival at 12 months of age in WD. Six of the nine treated WD patients (67%) met this primary outcome [16]. After one year, patients exhibited improvement in weight, gastrointestinal symptoms, and markers of liver dysfunction. A mid-term follow-up of these patients, and a second trial including 10 patients (LAL-CL08), was recently published [17]. The median age of surviving patients was 5.2 years and 3.2 years in the two trials, respectively. The clinical and biological benefits were confirmed with a mean follow-up of 46 months, but long-term data are lacking.

We report here the longest follow-up to date, of 5 WD patients treated with sebelipase alfa for a median duration of 7 years (1–10). Diagnostic features are described, as well as clinical (growth, survival and health-related quality of life (HRQoL), and biological data (liver function tests, plasma lipid profile and fat-soluble vitamin levels).

## Methods

### Study design and data collection

This retrospective study enrolled five WD patients who received sebelipase alfa on the long-term in France. WD was confirmed by LAL enzyme activity testing and *LIPA* gene mutational analysis (Table 1). Patients with LT or HSCT were excluded. We collected demographics, clinical and family history, method of diagnosis, physical and radiological examination, chemistry results, diet, medications and details of sebelipase alfa treatment (starting date, dose and side effects).

**Table 1:** Demographics, and biochemical and molecular  characteristics

	Patient 1	Patient 2	Patient 3	Patient 4	Patient 5
Consanguinity	−	+	+	+	+
Ethnicity	France-North Africa	Ivory Coast	Maroc	Turkey	Eritrea
Lymphocytes	Vacuolated	n.a	Vacuolated	Vacuolated	n.a
LIPA activity					
On WBC	5.1 nmol/h/mg (control: 31.2)	57 µmol/h/g (normal value: 350–2000)	n.a	n.a	n.a
On DBS (nmol/punch/h)	n.a	n.a	0	0	0
*LIPA* pathogenic variant					
Maternal allele	c.481delA	c.676-2A>G	c.429-1G>C	c.419G>C	c.260G>T
Paternal allele	c.538G>A	c.676-2A>G	c.429-1G>C	c.419G>C	c.260G>T
Variant impact					
Maternal allele	p.(Asn161Ilefs*19)	Disrupting splice acceptor site of intron 6	Disrupting splice acceptor site of intron 4	p.(Trp140Ser)	p.(Gly87Val)
Paternal allele	p.(Gly180Ser)	Disrupting splice acceptor site of intron 6	Disrupting splice acceptor site of intron 4	p.(Trp140Ser)	p.(Gly87Val)

Reference sequence NM_000235.4. *n.a.* not available, *WBC* white blood cells, *DBS* dried blood spot

Height, weight, head circumference and body mass index (BMI) plotted on growth curves are presented as standard deviation (SD) [18]. HRQoL is evaluated by the Pediatric Quality of Life Inventory questionnaire (PedsQL 4.0) [19]. On the questionnaire, the item scores range from 0 (better) to 4 (poorer). For analysis purposes, the scores are converted to a scale from 0 (poorer, = 4) to 100 (better, = 0).

### Statistical analysis

One-tailed Mann–Whitney tests with confidence intervals of 95%, comparing the pretreatment period with the sebelipase alfa period, were performed using GraphPad Prism v5.02 for Windows (GraphPad Software). *P* values < 0.05 were considered significant. Publisher (Office 365, Microsoft) was used to draw the figures.

## Results

### Patients

The five patients are described in Table 2. The early death of a previous sibling allowed a diagnosis before 1 month of age in three of them (Patients 2 to 4), who were also less severely ill at diagnosis.

**Table 2:** Patients' characteristics

	Patient 1	Patient 2	Patient 3	Patient 4	Patient 5	Median (min–max) or proportion
Age at diagnosis (months)	2	0	0	0	2	0 (0–2)
Follow-up (months)	120	83	37	84	14	83 (14–120)
Gender	Male	Male	Female	Female	Female	
Family history	−	+	+	+	−	3/5
Clinical features at diagnosis						
Diarrhea	+	+	−	+	+	4/5
Vomiting	+	+	+	−	+	4/5
Failure to thrive	+	+	+	−	+	4/5
Hepatomegaly	+	+	+	+	+	5/5
Splenomegaly	+	−	−	+	+	3/5
Biology at diagnosis						
Cytopenia	+	−	−	−	+	2/5
Elevated liver enzymes	+	+	−	−	+	3/5
Hypertriglyceridemia	+	−	−	−	+	2/5
Hypercholesterolemia	−	−	−	−	−	0/5
Radiological status						
Antenatal ultrasound anomalies	−	+	−	+	−	2/5
Bilateral adrenal calcifications	+	+	+	+	+	5/5
Thoracic lymphadenopathy	+					1/1
Abdominal lymphadenopathy	+	+	+	+	+	5/5
Diet						
Low in LCFA	+	+	+	+	+	5/5
Low in cholesterol	−	−	−	−	−	0/5
Enriched in MCT (at diagnosis)	+	+	+	+	+	5/5
Enriched in MCT (at last FU)	−	−	+	+	+	3/5
NG tube (at diagnosis)	−	+	−	+	+	3/5
NG tube (at last FU)	−	−	−	−	+	1/5
Treatment						
Cholestyramine	+	+	+	+	+	5/5
Ezetimibe	−	−	−	−	−	0/5
Statine	−	−	−	−	−	0/5
Fibrate	−	−	−	−	−	0/5
Vitamine A	+	+	+	−	−	3/5
Vitamine D	+	+	+	+	+	5/5
Vitamine E	+	+	+	+	+	5/5
Vitamine K	+	−	−	−	+	2/5
Sebelipase alfa						
Age at first dose (months)	4	1	0	1	2	1 (0–4)
Maintenance dose (mg/kg/dose)	5	3	3	3	5	3 (3–5)
Frequency (/ X weeks)	2	1	1	1	1	1 (1–2)
Treatment duration (months)	116	82	37	83	12	82 (12–116)
Venous access						
CVAD (number)	2	6	4	2	3	3 (2–6)
Last CVAD use (month)	65	66	26	n.a	n.a	65 (26–66)
At last FU	PVC	PVC	PVC	CVAD	CVAD	

*NG* nasogastric, *FU* follow-up, *LCFA* long-chain fatty acids, *MCT* medium-chain triglycerides, *CVAD* central venous access device, *n.a.* not applicable, *PVC* peripheral venous catheter

At last follow-up, sebelipase alfa was administered at a median maintenance dose of 3 mg/kg once a week. To reduce the frequency of the infusions, a trial of fortnightly infusions was made in Patient 1, with roughly twice the weekly dose (5 mg/kg/2 weeks). This was clinically and biologically well supported. The ERT was well tolerated on the long-term in all patients. Patient 5 experienced an anaphylaxis reaction during the first infusion, justifying hydroxyzine and betamethasone administration before the other infusions. Patients 1, 2 and 4 were initially included in the LAL-CL03 clinical trial (NCT01473875). Veinous access was challenging, especially in Patient 2, who required 6 central venous access devices, because of device infection or failure.

Abdominal lymphadenopathy was seen on ultrasound in Patient 1, and mediastinal lymphadenopathy on CT-scan. Biopsy of a lymph node showed foam cells. Afterwards, we found abdominal lymphadenopathy in all patients.

At diagnosis, all patients received a fat-free formula enriched with medium-chain triglycerides (MCT, 25–30% of total energy intake (TEI)). MCT enrichment was progressively decreased to 15% of TEI and even stopped in Patients 1 (3 years ½) and 2 (6 years ½). The diet was then liberalized with low fat milk and biscuits, but long-chain fatty acid restriction was maintained. Three patients required nasogastric tube feeding that could be suspended for two of them, Patients 2 and 4, at the age of 6 months and 2 years, respectively.

### Biological outcomes

**Fig. 1:**
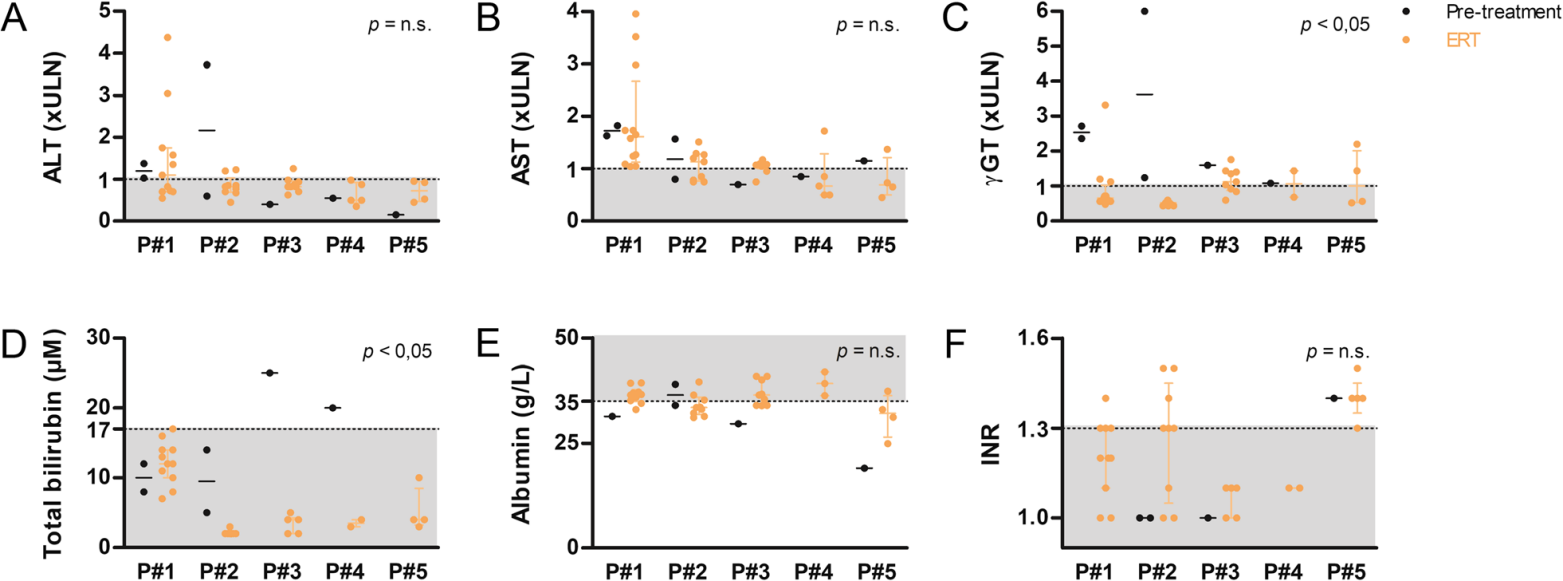
Liver function tests remained in the near-normal range in five Wolman disease (WD) patients treated with sebelipase alfa. **A** Alanine transaminase (ALT), **B** aspartate transaminase (AST), **C** gamma-glutamyltranspeptidase (γGT), **D** total bilirubin, **E** albumin and **F** international normalized ratio (INR) measured in Patients 1–5 (P#1–P#5), before (black) and under (orange) enzyme replacement therapy (ERT). ALT, AST and γGT are expressed as number of times of the upper limit of the normal (xULN). The grey zones and the dotted lines represent the normal values and their limits, respectively. Mann–Whitney test pre *versus* post, median ± interquartile range, *n.s.* not significant

WD leads to chronic liver injury along with alteration of the biological parameters related to liver function. Under sebelipase alfa treatment, alanine transaminase, aspartate transaminase, gamma-glutamyltranspeptidase (γGT), total bilirubin and albumin showed a trend to improvement in all patients (Fig. 1). Statistical significance was reached for γGT and total bilirubin. At last follow-up, none of the liver parameters were normal in all patients.

**Fig. 2:**
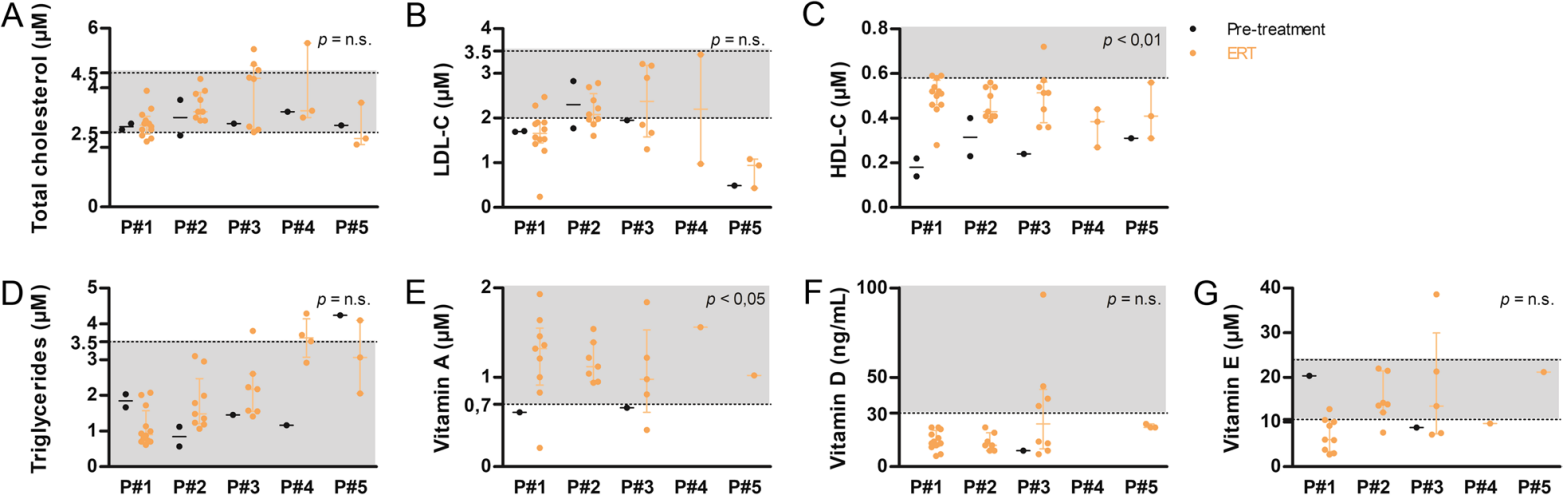
Long-term follow-up of cholesterol fractions, triglycerides and fat-soluble vitamin levels in five Wolman disease (WD) patients treated with sebelipase alfa. **A** Total cholesterol, **B** low-density lipoprotein cholesterol (LDL-C), **C** high-density lipoprotein cholesterol (HDL-C), **D** triglycerides, **E** vitamin A, **F** vitamin D, **G** vitamin E levels measured in Patients 1–5 (P#1–P#5), before (black) and under (orange) enzyme replacement therapy (ERT). The grey zones and the dotted lines represent the normal values and their limits, respectively. Mann–Whitney test pre *versus* post, median ± interquartile range, *n.s.* not significant

Alteration of the circulating cholesterol fractions and triglycerides is a key feature in WD. After sebelipase alfa treatment initiation, high-density lipoprotein cholesterol (HDL-C) improved significantly (*p* < 0.01) but did not normalize (Fig. 2). The impact of the treatment on total cholesterol, low-density lipoprotein cholesterol and triglycerides levels was less clear and did not reach significance.

In addition, we followed fat-soluble vitamin plasma levels to evaluate the impact of the treatment on their absorption. All patients received fat-soluble vitamins supplements (Table 2). Vitamin A levels normalized (or were normal at last follow-up) in all patients under sebelipase alfa. The ERT effect on vitamins D and E levels was less clear despite supplementation prescribed to the patients (Fig. 2). No patient exhibited clinical consequences associated with fat-soluble vitamin deficiency.

### Clinical outcomes

**Fig. 3:**
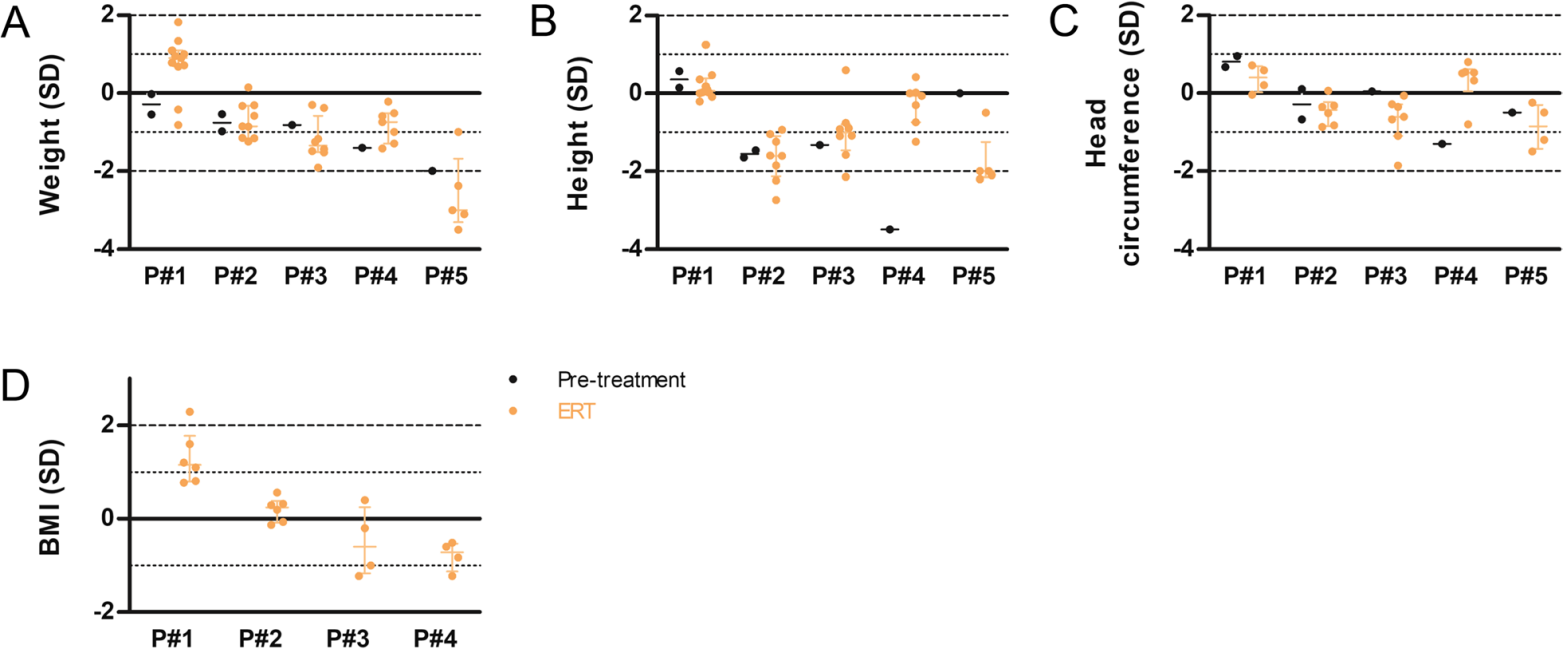
Long-term follow-up of growth parameters in five Wolman disease (WD) patients treated with sebelipase alfa. **A** Weight, **B** height, **C** head circumference and **D** body mass index (BMI) expressed in standard deviations and measured in Patients 1–5 (P#1–P#5), before (black) and under (orange) enzyme replacement therapy (ERT). BMI growth curves start at 2 years explaining the absence of values in P#5 and before treatment. Median ± interquartile range

The natural history of WD is associated with failure to thrive, diarrhea, vomiting and hepatosplenomegaly. At last follow-up, all patients had growth parameters (weight, height, head circumference and BMI) above − 2 SD (Fig. 3). Under ERT, digestive symptoms (diarrhea and vomiting) resolved in all patients. Patients reported recurrence of greasy diarrhea in case of diet deviation (high-fat foods). All patients exhibited objective chronic abdominal distention (probably related to fat accumulation in the digestive wall and the mesentery) but they had no complaint related to that (i.e. no bloating nor chronic abdominal pain). Hepatosplenomegaly disappeared in all patients except in Patient 5 (12 months after ERT initiation).

Nasogastric feeding tube is still required in Patient 5 because of food aversion persisting 12 months after the start of ERT. Patient 5 also required parenteral nutrition at home after an episode of acute gastroenteritis. None of the patients had long-term parenteral nutrition.

**Fig. 4:**
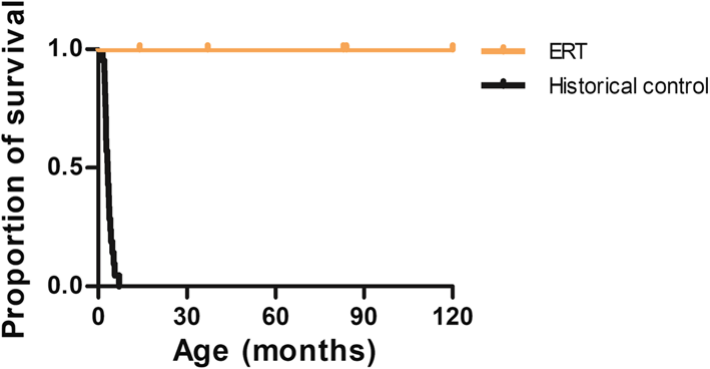
All the Wolman disease (WD) patients treated with sebelipase alfa survived in our cohort. Proportion of survival in WD patients treated with enzyme replacement therapy (ERT, n = 5) compared with WD patients from a historical cohort (LAL-1-NH01 [16], n = 21)

All the patients were alive at the moment of the publication (100% survival, Fig. 4). Patients 2 and 3 had a previously affected sibling who died at the age of 4 months because of WD complications (liver failure and malnutrition). Patient 4’s affected sibling underwent HSCT in a research setting. He died immediately after the procedure at the age of 5 months.

We used the PedsQL questionnaire [19] to assess the HRQoL of the patients at last follow-up. Both parents and patients (when applicable) reported acceptable or high HRQoL globally and in all 4-dimensional scales (Table 3). Cognitive development was normal and no patient had special education needs.

**Table 3:** PedsQL scores

	Patient 1 (%)	Patient 2 (%)	Patient 3 (%)	Patient 4 (%)	Patient 5 (%)
Patient evaluation	71	61	n.a	80	n.a
Physical functioning (8 items)	75	56	n.a	88	n.a
Emotional functioning (5 items)	60	60	n.a	90	n.a
Social functioning (5 items)	70	70	n.a	70	n.a
School functioning (3 or 5 items)	75	60	n.a	70	n.a
Parental evaluation	82	51	85	85	100
Physical functioning (8 items)	75	47	84	91	100
Emotional functioning (5 items)	80	75	70	80	100
Social functioning (5 items)	85	45	100	100	100
School functioning (3 or 5 items)	90	40	n.a	65	n.a

*n.a.* not applicable

Patients 1 and 2 underwent electromyography because of footdrop gait and waddling walk, respectively. The testing was normal in both patients. Patient 4 had muscle fatigue when walking and her muscle strength testing was normal. They all showed spontaneous recovery of their muscular weakness.

## Discussion

WD is an ultrarare disease leading to death before 12 months of age if untreated [8]. Sebelipase alfa ERT trials reported 68% survival at 5 years [17] but long-term follow-up in real-life settings are lacking. Here, we report the bio-clinical evolution of 5 WD patients treated with sebelipase alfa up to 10 years and we confirm its strong efficacy. ERT allowed WD patient survival far beyond the prognosis experienced in the pre-ERT era. All patients survived with a median follow-up of 7 years, and they reported satisfactory HRQoL. Liver function tests, lipid profiles and growth parameters (nearly) normalized under treatment.

All patients presented with strongly abolished LIPA activity and clinical manifestations before one month of age. DNA sequence analysis demonstrated 3 pathogenic variants (c.260G>T [5, 10, 17, 20, 21], c.481delA [22], c.676-2A>G [10, 23]) reported in patients presenting with LALD. *LIPA* variants of Patient 1 (c.538G>A) and Patient 3 (c.429-1G>C) were previously published in LAL-CL03 trial [16, 17] and in a large-scale screening of LALD in at risk population [24], respectively.

Most of our patients (4/5) experienced no medically relevant infusion-associated reaction (IAR) during the follow up, thus confirming the acceptable safety profile of the product [17]. One patient had one medically relevant IAR justifying systematic infusion premedication.The anaphylactic reaction presented by the patient might be favored by the absence of sebelipase alfa titration (i. e. the patient received a 5 mg/kg sebelipase alfa infusion as first dose compared to progressive increase of the dose for the 4 other patients). Our study might reflect the real-world IAR incidence during sebelipase alfa ERT compared to the clinical trial IAR incidence comprised between 56% [16] and 80% [17].

Longitudinal follow-up from birth allowed us to compare biological status before and under ERT. Most biological parameters improved under ERT and some of them normalized but only γGT, total bilirubin, HDL-C and vitamin A reached statistical significance. This might be explained by small sample size and the near normal biological values before ERT considering the early diagnosis (median age: 3 weeks) and the prompt ERT initiation (median age: 7 weeks) in 3 patients. Phase 2/3 clinical trials evaluating sebelipase alfa in WD patients gave similar biological results [16, 17].

None of our patient exhibited lack of clinical response to ERT that could not be alleviated by sebelipase alfa dose adjustment. In LAL-CL08, 3 patients (30%) exhibited lack of clinical response related to anti-drug antibody [17]. Interestingly, these 3 patients were homozygous for *LIPA* whole gene deletion which could explain the strong immunological response directed against the recombinant enzyme. None of the WD patients included in this study harbored such deletion.

The 100% survival in our cohort contrasts with the survival rate of 55% and 80% in the LAL-CL03 [16] and LAL-CL08 [17] trials, respectively. Our good results can be explained by the high proportion of positive family history (3/5) in our cohort leading to early diagnosis and better clinical status at ERT initiation. Other explanations might be the evolution in the understanding of disease management [17] and the awareness of the clinicians to early disease recognition (also in absence of family history, like in Patient 5) since sebelipase alfa got marketing authorization [7].

Three patients exhibited a neuromyopathic phenotype (footdrop gait, waddling walk or muscle fatigue) under sebelipase alfa treatment that resolved spontaneously. As seen in ERT for Pompe disease [25], clinicians must be alert to the development of attenuated (or new) phenotype in WD patients treated with ERT. For example, fat accumulation in large abdominal (and thoracic) lymphadenopathy, in the digestive wall and in the mesentery persisted in WD patients treated with sebelipase alfa showing that the disease is not cured. Yet, normal neurodevelopmental evolution during sebellipase alfa clinical trials [17] and high HRQoL reported in our cohort are encouraging.

Sebelipase alfa allowed a normal growth and a good quality of life on the long-term in this cohort (up to 10 years). Hopely, it will add evidence that sebelipase alfa is a cost-effective treatment for WD. In 2018, the National Centre for Pharmacoeconomics (NCPE, Ireland) [26] and the Canadian Agency for Drugs & Technologies in Health (CADTH) [27] concluded that the estimated cost effectiveness of sebelipase alfa for WD was low (NCPE) and/or impeded by substantial uncertainty, mainly regarding the long-term efficacy (CADTH). Based on the same conclusion, the National Institute for Health and Care Excellence (NICE, United Kingdom) did not recommend sebelipase alfa reimbursement for WD. In France, the Haute Autorité de Santé (HAS) did recommend reimbursement for WD but not for CESD.

In its report, the NICE suggested that sebelipase alfa is a potential bridging therapy to HSCT in WD. During the pre-ERT era, the survival rate after HSCT for WD was 25% (3/12 patients) [11–15]. The reasons for this poor outcome are multifactorial but comprise mainly the critical condition of the patients before HSCT and the need for a more aggressive conditioning regimen [12]. We looked for hematopoietic stem cell donor for two patients in our cohort in order to plan a HSCT once their clinical condition was stabilized under ERT. Unfortunately, unrelated matched donor could not be found. Vijay et al.[17] reported 2 patients treated with sebelipase alfa which underwent HSCT around two years of age in order to manage the loss of clinical efficacy resulting from high ADA titers. After the procedure, the clinical efficacy recovered at a reduced sebelipase alfa dose. There is no mention of graft failure or trial/success to stop the ERT thanks to the procedure [17].

As reported previously [28], and confirmed in this report, early WD diagnosis enables precocious ERT initiation and better clinical outcome. Optimized methods are available to perform WD screening on dried blood spot [29, 30]. This raise the question of expanding the newborn screening programs to WD [31].

To the best of our knowledge, this report is the longest follow-up of WD patients treated with sebelipase alfa. It is also the first time that the HRQoL of the patients and their parents is evaluated. In order to improve HRQoL, we are planning to try fortnightly sebelipase infusion in the older patients (> 5–10 years, see Patient 1) along with home infusion therapy. The evolution of the liver and gastro-intestinal tract microscopical aspect under sebelipase alfa ERT (fibrosis, fat accumulation, villi aspect, …) remains unknown and will be reported (separately) very soon. Due to the rarity of the disease and the recent sebelipase alfa marketing authorization, the cohort only comprises 5 patients.

## Conclusion

Sebelipase alfa allowed 100% survival of 5 WD patients with near normal bio-clinical and growth parameters follow-up, up to ten years. Early diagnosis and treatment initiation were key features to reach good clinical outcomes. Very long-term follow-up (> 20 years) and HSCT in WD treated with sebelipase alfa have to be evaluated in the future.

## Data Availability

The datasets generated during and/or analysed during the current study are available from the corresponding author on reasonable request.
